# Is Particle Pollution in Outdoor Air Associated with Metabolic Control in Type 2 Diabetes?

**DOI:** 10.1371/journal.pone.0091639

**Published:** 2014-03-11

**Authors:** Teresa Tamayo, Wolfgang Rathmann, Ursula Krämer, Dorothea Sugiri, Matthias Grabert, Reinhard W. Holl

**Affiliations:** 1 Institute of Biometrics and Epidemiology, German Diabetes Center, Leibniz Center for Diabetes Research at Heinrich Heine University Düsseldorf, Düsseldorf, Germany; 2 Institute for Environmental Medicine (IUF), Leibniz Center at Heinrich Heine University Düsseldorf, Düsseldorf, Germany; 3 Institute for Epidemiology and Medical Biometry, University of Ulm, Ulm, Germany; Innsbruck Medical University, Austria

## Abstract

**Background:**

There is growing evidence that air pollutants are associated with the risk of type 2 diabetes. Subclinical inflammation may be a mechanism linking air pollution with diabetes. Information is lacking whether air pollution also contributes to worse metabolic control in newly diagnosed type 2 diabetes. We examined the hypothesis that residential particulate matter (PM_10_) is associated with HbA_1c_ concentration in newly diagnosed type 2 diabetes.

**Methods:**

Nationwide regional levels of particulate matter with a diameter of ≤10 µm (PM_10_) were obtained in 2009 from background monitoring stations in Germany (Federal Environmental Agency) and assigned to place of residency of 9,102 newly diagnosed diabetes patients registered in the DPV database throughout Germany (age 65.5±13.5 yrs; males: 52.1%). Mean HbA_1c_ (%) levels stratified for air pollution quartiles (PM_10_ in µg/m^3^) were estimated using linear regression models adjusting for age, sex, BMI, diabetes duration, geographic region, year of ascertainment, and social indicators.

**Findings:**

In both men and women, adjusted HbA_1c_ was significantly lower in the lowest quartile of PM_10_ exposure in comparison to quartiles Q2–Q4. Largest differences in adjusted HbA_1c_ (95% CI) were seen comparing lowest quartiles of exposure with highest quartiles (men %: −0.42 (−0.62; −0.23)/mmol/mol: −28.11 (−30.30; −26.04), women, %: −0.28 (−0.47; −0.09)/mmol/mol: −0.28 (−0.47; −0.09)).

**Interpretation:**

Air pollution may be associated with higher HbA_1c_ levels in newly diagnosed type 2 diabetes patients. Further studies are warranted to examine this association.

## Background

The associations between exposure to traffic-related air pollution and cardiovascular disease, cardiovascular hospital admission rates, and all-cause or cardiovascular mortality are well established [1], [2]. Patients with type 2 diabetes are more susceptible to these adverse effects [3]. Besides, traffic-related air pollution was associated with diabetes-associated mortality in a current study [4]. Recently, evidence is growing that air pollutants (nitrogen oxides (NOx), particulate matter (PM) with a diameter of ≤10 µm or 2.5 µm) may also be associated with type 2 diabetes prevalence and incidence [5]–[9]. Diabetes risk was increased by 4%–15% per interquartile range (IQR) of particulate matter with a diameter of ≤10 µm (PM10) [5], [7], by 25% per IQR increase in nitrogen oxides (NOx) [9], and by 11% for living in short distance (<50 m) to a major road [8].

Furthermore, in a cross-sectional Taiwanese study, higher HbA1c levels were observed with increased traffic-related air pollution in the general population [10]. To our knowledge, the association with HbA1c has not been examined in patients with type 2 diabetes so far. HbA1c is mainly used as an indicator for metabolic control in persons with type 2 diabetes. Guidelines stress the importance of a good metabolic control in most patients in order to prevent complications [11]. Even small increase in HbA1c due to worse metabolic control could affect long-term cardiovascular risk and mortality [12], [13]. However, air pollution may impede an optimal metabolic control by increased subclinical inflammation [14]. In addition, inflammatory processes may also increase the vulnerability to cardiovascular health effects (e.g. myocardial infarction) in persons with type 2 diabetes who are exposed to air pollution [5], [15].

Thus, we examined HbA_1c_ concentration in individuals with newly diagnosed type 2 diabetes and its association with residential air pollution in a large German cohort based on the DPV documentation system (Diabetessoftware für Prospektive Verlaufsbeobachtung) using data assessed in routine care [16].

## Methods

### Ethics Statement

Informed consent was obtained from every patient at each participating center (more than 300 GP practices, hospitals, rehabilitation clinics). The consent procedure and documentation (either verbal or written depending on institution) was approved by local institutional review boards or the responsible commissioners for data protection of participating centers. The locally collected study data was anonymized before transferal to the data management center at Ulm University. The DPV study and the consent and data collection procedures were approved by local data control authorities and the institutional review board at Ulm University.

### Study Population

Patients with newly diagnosed type 2 diabetes aged 18 years or older who were registered between 2005 and 2009 in the DPV database were selected for the analysis. The DPV database covers anonymized data on more than 200,000 patients from 336 participating health facilities such as diabetologists, primary care practices, hospitals and rehabilitation clinics in Germany [16]. Nationwide, physicians from participating centers document each patient with diabetes diagnosis and data e.g. on age, sex, diabetes duration, HbA_1c_, laboratory measurements and medication. After informed consent, patient data are transferred electronically twice a year to the documentation center in Ulm. For this purpose, a computer software was installed in participating centers that serves for medical documentation in routine care as well as for data collection in the DPV study with considerable overlap between both functions.

For the present study, the data was analyzed cross-sectionally. Only patients with a diabetes duration of a maximum of 2.5 years (range: 0.5–2.5 years) were included in order to examine a homogeneous patient group with doctor’s visits in a comparable time frame in which similar treatment options were available. Of 14,042 patients, 1,984 individuals had missing information on body mass index (BMI) or HbA_1c_. Furthermore, we restricted the sample to persons treated in ambulatory care units of hospitals to examine a more homogeneous patient group with better documentation. Thus, 9,102 participants were available for analysis.

### Measurements

HbA_1c_ measurements were measured locally and adjusted to the Diabetes Control and Complications Trial (DCCT) normal range using the multiple of the mean method based on the reference range for healthy subjects in each laboratory [16], [17].

Nationwide regional levels of particulate matter with a diameter of ≤10 µm (PM_10_) were obtained for Germany based on a raster with a cell size of eight kilometer × eight kilometer. These maps were generated by the environmental agency of Germany “Umweltbundesamt II 4.2” (monitoring of air quality) using the chemical REM-CALGRID^2^ (RCG) model into which PM10 measured at background monitoring stations was integrated [18]. The REM-CALGRID^2^ itself has been used since 1999. It is fitted with meteorological and PM time-series data of 150 German monitoring stations and additional data from other European countries. The integration of PM10 for 2009 from the monitoring sites was done using the optimal interpolation method (OI). This model includes inhomogeneous spatial auto-covariance between PM10 from monitoring stations and the broad scale background information for representative (reference) areas. Different models of covariance were applied for each calculated rasterpoint depending on monitored and modeled PM_10_. Suburban sites were overrepresented in monitoring stations. Therefore, prior to integration, PM_10_ measurements from monitoring stations were corrected for this suburban/rural bias. Based on this approach, we calculated the annual PM_10_ for each five-digit postcode area (100 areas) by intersection of the German PM_10_ raster with the German postcode map. Each postcode area obtained an area-weighted mean of PM10 of included rastercells. Intersection was done with ArcGIS version 9, Environmental Systems Research Institute (ESRI), California, USA.

Data of the interpolation raster are leveled to the measurement range of background monitoring stations. The measurements are further leveled out by integration by interpolation. In other words, data smoothing is needed to characterize the average pollution level in each raster area which includes measurements from e.g. urban and rural sites with heterogeneous levels of PM_10_. This was accomplished by interpolation over the raster cells and furthermore by integration to calculate the mean pollution of each raster.

Additionally, patient’s residency was available on postcode level only. Depending on population density, postcode areas may extend to dozens of km^2^ in regions with low population density. As a consequence, the interpolation raster is further coarsened to area-weighted averages for each postcode area. Therefore measurements are not precise for the place of residence but leveled to the area around it. However, people usually do not stay at their place of residence throughout the day but change their position for work or leisure time. Hence, this approach yields a rather valid estimation of annual background exposure for patients who mostly stay within the range of some kilometers around their place of residence. Further information on the application of the REM-CALGRID^2^ model has been described elsewhere [19].

Height and weight were measured during doctors’ visits. Hypertension and dyslipidemia were defined according to doctor’s diagnosis or disease-specific medication.

As social indicators we included formal schooling (no high school diploma, yes/no) or immigrant status (yes/no).

### Geographic Location of Nielsen Areas

Nielsen areas were first used in market research to determine geographic regions sharing characteristics of federal economy and consumer behavior (The Nielsen Company, NY, USA). In Germany, seven Nielsen areas are distinguished each of which includes one or more coherent federal states. Germany consists of sixteen federal states with autonomous jurisdiction in many aspects of administrative law affecting e.g. health politics and education. Some of the German federal states are rather small (Bremen and Hamburg). In these smaller federal states, only some physicians participated in the DPV study, so that numbers in some regions were low and protection of data privacy was not guaranteed on federal state level. Therefore, Nielsen areas were used to allow for adjustment of regional disparities in health care and of geographic features. Because Nielsen areas are characterized by economic factors they also function as an area-based social indicator reflecting e.g. unemployment rate of a region.

Nielsen area 1: Hamburg, Bremen, Schleswig-Holstein, Lower Saxony (North)

Nielsen area 2: North Rhine-Westfalia (West)

Nielsen area 3 Hesse, Rhineland-Palatinate, Saarland, Baden-Württemberg (Southwest)

Nielsen area 4 Bavaria (South)

Nielsen area 5 Berlin (Northeast)

Nielsen area 6 Mecklenburg-Vorpommern, Brandenburg, Saxony-Anhalt (Northeast)

Nielsen area 7 Thuringia, Saxony (East)

### Statistical Analysis

For descriptive analyses, mean (SD) were calculated for continuous variables and proportions for categorical variables. Quartiles of exposure to PM_10_ (µg/m^3^) in 2009 were calculated using the distribution in the study population. Mean (adjusted) HbA_1c_ levels stratified for air pollution quartiles (PM_10_ in µg/m^3^) and mean difference in HbA_1c_ levels between quartiles and corresponding 95% confidence intervals were estimated using generalized linear regression modeling adjusting for age, sex, BMI, diabetes duration, geographic region (Nielsen area), year of ascertainment, and the social indicator (no high school diploma or immigration background). In addition, analyses stratified by sex were performed. For sensitivity analyses, models were fitted including both patients treated in ambulatory care units of hospitals and those treated in outpatient practices of general practitioners and diabetologists. Furthermore, the linear association of continuously measured PM_10_ (µg/m^3^) with HbA1c measurements was analyzed. Moreover, age- and sex- adjusted models were fitted, additionally adjusting for types of medication (oral anti-diabetic medication (OAD) or insulin) and co-morbidities (hypertension or dyslipidaemia). The limit of statistical significance was set at p<0.05. Statistical analyses were carried out with SAS for Windows version 9.3. (SAS Institute, Cary, NC, USA).

## Results

The studied sample comprised 9,102 patients (4,356 women, 4,746 men) with newly diagnosed type 2 diabetes whose mean diabetes duration was 1.5 years (SD 0.6 years).

Of all patients, 49% were registered in the South (Nielsen regions 3 and 4), 30% in the West (Nielsen 2), 16% in the North-East (Nielsen 5–7) and 5% in the North of Germany (Nielsen 1). In Table 1 characteristics of the study participants are shown. On average, the sample was mostly elderly and obese. Numbers of participants who had left school without a high school diploma or who had immigrant status (social indicator) were low (n = 182). The mean annual HbA_1c_ (%) was 7.2 (SD: 1.9%). Overall, more than one third was treated with insulin, and more than half of the sample received oral glucose-lowering drugs. Hypertension or antihypertensive drug prescriptions were found in approximately two thirds. Patients from different geographic Nielsen areas differed in several aspects (Table 2). Patients from the South and West were slightly older on average, had lower HbA1c levels and a lower mean BMI especially in comparison to patients from the East, Northeast and Berlin. Exposure to PM_10_ was higher in the densely populated areas of Berlin and the Rhine-Ruhr-Area (West). Medical treatment also differed considerably between regions with a very high percentage of patients receiving oral anti-hyperglycaemic drugs and a low percentage receiving insulin in the South and a reversed pattern in Berlin and the Northeast.

**Table 1:** Characteristics of participants with type 2 diabetes*.

	Total	Women	Men
Number of participants (N)	9,102	4,356	4,746
Age at baseline examination (years)	65.5 (13.5)	67.3 (14.0)	63.9 (12.9)
Diabetes duration (years)	1.5 (0.6)	1.5 (0.6)	1.5 (0.6)
HbA_1c_ (%)	7.2 (1.9)	7.1 (1.8)	7.3 (2.0)
Body mass index (kg/m^2^)	30.6 (6.4)	30.9 (6.9)	30·4 (5.9)
Mean PM10 year 2009 (µg/m^3^)	19.6 (4.3)	19.7 (4.2)	19·6 (4.3)
Hypertension/antihypertensive treatment (%)	65.2	64.7	65.6
Dyslipidaemia/lipid-lowering treatment (%)	66.8	67.2	66.5
Insulin treatment (%)	35.3	33.9	36.6
Oral antihyperglycaemic drugs (%)	57.1	55.6	58.5

*Results are numbers (N), frequencies in % or means (SD). HbA1c levels in % (NGSP) can be converted to mmol/mol (IFCC) by application of the following formula: IFCC = (10.93*NGSP)−23.50.

**Table 2:** Characteristics of participants with type 2 diabetes per geographic region (Nielsen area).

	1 (N)	2 (W)	3 (SW)	4 (S)	5 (B)	6 (NE)	7 (E)	P-value
Number of participants (N)	451	2729	3050	1451	412	850	159	
Women (N)	176	1344	1435	706	215	409	71	0.002
Age at baseline examination (years)	64.8 (12.4)	67.6 (12.8)	64.1 (14.0)	66.2 (13.2)	63.0 (13.2)	64.9 (13.9)	63.8 (15.4)	<0.0001
Diabetes duration (years)	1.5 (0.6)	1.5 (0.6)	1.5 (0.6)	1.5 (0.6)	1.4 (0.6)	1.5 (0.6)	1.5 (0.6)	0.438
HbA_1c_ (%)	7.4 (2.7)	7.1 (1.8)	7.2 (1.9)	7.2 (1.9)	7.4 (1.9)	7.6 (2.0)	7.1 (1.7)	<0.0001
Body mass index (kg/m^3^)	30·4 (5.7)	30·2 (6.2)	30·8 (6.4)	30·3 (6.5)	31·5 (6.8)	31.2 (6.5)	31.6 (6.3)	<0.0001
Mean PM10 year 2009 (µg/m^3^)	15.9 (2.1)	23.5 (3.8)	17.5 (2.9)	17.7 (2.1)	25.0 (1.7)	17.1 (2.5)	19.1 (1.5)	<0.0001
Hypertension/antihypertensive treatment (%)	73.4	53	68.7	76.6	68.4	67.1	61	<0.0001
Dyslipidaemia/Lipid-lowering treatment (%)	60.5	66.6	67.4	71.3	51.9	70.7	54.7	<0.0001
Insulin treatment (%)	25.1	29.8	38.7	28.5	41.7	54.5	39	<0.0001
Oral antihyperglycaemic drugs (%)	56.3	55.6	54.4	71.1	54.6	49.1	60.4	<0.0001

Results are numbers (N), frequencies in % or means (SD). Abbreviation of Nielsen areas: 1 (N) = Hamburg, Bremen, Schleswig-Holstein, Lower Saxony (North); 2 (W) = North Rhine-Westfalia (West); 3 (SW) = Hesse, Rhineland-Palatinate, Saarland, Baden-Württemberg (Southwest); 4 (S) = Bavaria (South); 5 (B) = Berlin (Northeast); 6 (NE) = Mecklenburg-Vorpommern, Brandenburg, Saxony-Anhalt (Northeast); 7 (E) = Thuringia, Saxony (East). HbA1c levels in % (NGSP) can be converted to mmol/mol (IFCC) by application of the following formula: IFCC = (10.93*NGSP)−23.50.

Table 3 shows adjusted differences in HbA_1c_ levels and corresponding 95% confidence intervals across particle exposures. HbA_1c_ (%) was significantly lower in both men and women in the lowest quartile of PM_10_ exposure in comparison to quartiles with higher levels of exposure (Q1 vs. Q2–Q4). Men in Q2 and Q3 also had substantially lower adjusted HbA_1c_ levels than those in the highest quartile of PM_10_ exposure (Q2 vs. Q4: −0.23; 95% CI: −0.44, −0.02/Q3 vs. Q4: −0.25; −0.43, −0.06).

**Table 3:** Adjusted mean HbA_1c_ and difference in HbA_1c_ comparing quartiles of particulate matter (PM_10_) exposure in type 2 diabetes patients*.

Quartiles of exposure	Total sample	Women	Men
	N = 9,102	N = 4,356	N = 4,746
**Mean adjusted HbA_1c_ in %**			
Q1<16.40 µg/m^3^	6.9	6.7	7.2
Q2 16.40−<18.05 µg/m^3^	7.1	6.9	7.4
Q3 18.05−<21.10 µg/m^3^	7.1	6.9	7.4
Q4≥21.10 µg/m^3^	7.3	6.9	7.6
**Difference in HbA_1c_ levels in %**			
**Q1 vs. Q2**	−**0.20 (**−**0.33,** −**0.08)**	−**0·22 (**−**0.38,** −**0.05)**	−**0.20 (**−**0.37,** −**0.02)**
**Q1 vs. Q3**	−**0·21 (**−**0·32,** −**0·09)**	−**0·24 (**−**0·39;** −**0·08)**	−**0·18 (**−**0.34;** −**0.02)**
**Q1 vs. Q4**	−**0·36 (**−**0·49,** −**0·22)**	−**0·28 (**−**0·47,** −**0·09)**	−**0·42 (**−**0.62,** −**0.23)**
**Q2 vs. Q3**	0·00 (−0.13, 0.12)	−0·02 (−0.19; 0.15)	0·02 (−0.16, 0.20)
**Q2 vs. Q4**	−**0.15 (**−**0.30,** −**0.01)**	−0.07 (−0.26; 0.13)	−**0.23 (**−**0.44,** −**0.02)**
**Q3 vs. Q4**	−**0.15 (**−**0.28,** −**0.02)**	−0·04 (−0.21, 0.13)	−**0.25 (**−**0.43,** −**0.06)**

*Results are adjusted means for HbA_1c_ in % calculated from generalized linear regression models. Models were fitted adjusting for age, sex, body mass index, duration of diabetes, geographic region, year of treatment, and social indicators (low education, immigration background). Furthermore, difference in HbA_1c_ levels in % (95% CI) comparing quartiles of PM_10_ exposure also derived from linear regression models are presented. Group differences are considered as significant (highlighted in bold) if corresponding 95% confidence intervals do not include 0. HbA1c levels in % (NGSP) can be converted to mmol/mol (IFCC) by application of the following formula: IFCC = (10.93*NGSP)−23.50.

In the adjusted model, further variables associated with HbA_1c_ were sex, BMI, age, diabetes duration and geographic area (Nielsen areas). We did not observe any association with the social indicator variable (p = 0.47). Of note, crude mean HbA1c values changed after adjustment. Adjusting for age and sex only, HbA1c levels were significantly lower in quartile 1 than in all other quartiles (e.g. Q1: 7.1%; Q4: 7.3%). With further adjustment for BMI, diabetes duration and region (Nielsen area), differences in HbA1c levels were more pronounced.

While the main analysis (see tables) included only patients treated in ambulatory care units of hospitals, we carried out a sensitivity analysis encompassing both patients treated in hospitals and in practices of general practitioners. In this sample of 12,058 participants, the differences between quartiles of pollution were slightly attenuated in the final model adjusted for age, sex, BMI, diabetes duration (years), Nielsen areas, year of treatment, institution of treatment (GP yes/no) and the social indicator. Overall, mean adjusted HbA1c levels were 0.2–0.4% (2–4 mmol/mol) lower in all quartiles. Difference in Quartiles Q1 and Q3 did not reach statistical significance (−0.12; 95% CI −0.25, 0.01) while the overall tendency of all other group comparisons remained similar.

In further models also adjusted for age, sex, BMI, diabetes duration (years), Nielsen areas, year of treatment and the social indicator but fitted with the continuous measurements of PM10 confirmed a significant association with HbA1c (estimate: 0.025, SE 0.007, p = 0.0001). With further adjustment for clinical information such as treatment with oral anti-hyperglycemic drugs only (yes/no), hypertension or anti-hypertensive drugs, dyslipidaemia or lipid-lowering drugs results hardly changed. Inclusion of the information on treatment with insulin (yes/no) attenuated the association with air pollution, but it remained significant (0.014, SE 0.002, p = 0.02).

## Discussion

The novel finding of the present study is that exposure to particulate matter (PM_10_) is associated with higher HbA_1c_ levels (worse metabolic control) in newly diagnosed type 2 diabetes patients. Our findings are in line with previous results of a population-based Taiwanese study where HbA_1c_ levels increased by 1.4% (95% CI 1.1–1.7) for each inter-quartile range increase in PM_10_ pollution.^10^ In our study in type 2 diabetes, HbA_1c_ increase was less pronounced, however, on substantially lower levels of air pollution exposure. Adjusting for insulin attenuated the association but it remained significant. This has two implications: first, patients living in highly polluted areas would possibly require insulin at an earlier stage of their disease, and second, metabolic control is impaired in these patients, even under early medication with insulin and they would possibly require higher dosages of insulin to achieve HbA_1c_ targets of guideline recommendations. However, further studies are warranted to corroborate these hypotheses.

Despite the relatively small increase in HbA_1c_ levels for each quartile increment of particulate matter exposure in our study, this difference might contribute to a considerable long-term increase of micro- and macrovascular complications. In the population-based Rancho Bernardo study, a 1% increase in HbA_1c_ was associated with a hazard ratio for cardiovascular mortality of 1.26 (95% CI 1.03–1.55) even at non-diabetic levels [20].

Further evidence on the importance of comparatively low levels of air pollution comes from another recent study [21]. In 25 otherwise healthy individuals with impaired glucose tolerance from rural areas, even small increases in PM_2.5_ concentrations affected insulin resistance after short periods and at small levels of exposure (5 days). Furthermore, in a Swedish study, nitrogen oxide exposure at levels below current WHO air quality guidelines during pregnancy was associated with gestational diabetes and pre-eclampsia [22].

Rajagopalan and Brook have summarized pathophysiologic pathways which are currently discussed to explain the association between air pollution and type 2 diabetes [15]. Among these, systemic inflammation and oxidative stress play an important role. This mechanistic pathway may also explain the association of air pollution with type 2 diabetes, gestational diabetes and gestational complications (e.g. pre-eclampsia) and moreover the deleterious cardiovascular effects of air pollution in patients with type 2 diabetes [3], [15], [23]. In response to inhaled pollutants a state of chronic systemic inflammation and oxidative stress occurs which subsequently may aggravate insulin resistance and trigger metabolic disturbances [24].

Data on possible pathophysiologic pathways have mostly been obtained from mouse models. These mouse models also suggested an interaction of PM_2.5_ exposure with high-fat diet during the development of metabolic disturbances [14]. However, mice fed normal chow also showed enlarged visceral fat contents and increased macrophage infiltration in visceral adipose tissue after exposure to PM_2.5_ for 10 weeks [25]. Another recent mouse model suggested further pathways linking air pollution with type 2 diabetes by showing that PM_2.5_ exposure had adverse effects on glycogen storage in the liver which led to the development of a NASH like phenotype [26].

As an example for the inflammatory response in human beings, short-term exposure with concentrated ambient particles induced mild pulmonary inflammation and increased plasma fibrinogen content in healthy volunteers [27]. However, it should also be noted that a recent cross-sectional study in elderly women found no convincing evidence for an association between exposure to PM_10_ and elevated plasma levels of proinflammatory biomarkers [28].

Thus, effects of air pollutants are suggested to affect various organs and systems of the body including glucose metabolism [3]. Given the world-wide burden of traffic- and industry-related air pollution, and of type 2 diabetes, pollution control might be very effective to lower the burden of disease. Strategies to reduce exposure to traffic related air pollution such as urban planning, land-use decisions and individual strategies need to be developed and tested.

### Limitations

First, HbA_1c_ levels were not centrally determined. In order to reduce between-laboratory variation, HbA_1c_ values were standardized to the Diabetes Control and Complication Trial Research Group reference range (DCCT) using the multiple of the mean method.^17^]. Furthermore, lifestyle factors (physical activity, nutrition) were not assessed. Also, detailed information on socioeconomic circumstances of patients was not available (e.g. schooling degree, income situation, professional career). Therefore, uncontrolled confounding by individual socioeconomic or lifestyle factors may have played a role in the association that we observed. Finally, there may be an uncertainty of measurements especially in some participants with a high mobility or unusually high exposures at work or indoors (e.g. due to open fires). Thus, the association of air pollution with HbA1c levels we found may actually be caused by socioeconomic or lifestyle factors we could not adjust for. However, the strength of the study is the use of a huge nationwide sample covering both rural and urban regions across Germany.

### Conclusions

Patients with type 2 diabetes exposed to higher levels of air pollution showed higher HbA_1c_ levels and consequently might be at a higher risk for complications. However, we cannot rule out residual confounding due to those socioeconomic or lifestyle factors that were not available for analysis. Considering the worldwide burden of type 2 diabetes and of air pollution, this association needs further corroboration.
